# The fallacy of meritocracy in the real-life social order

**DOI:** 10.3389/fsoc.2026.1766088

**Published:** 2026-03-09

**Authors:** Lucas Teles da Silva, Dimitri Marques Abramov

**Affiliations:** 1 Laboratory of Neurobiology and Clinical Neurophysiology, National Institute of Women's Children's and Adolescents' Health Fernandes Figueira, Oswaldo Cruz Foundation, Rio de Janeiro, Brazil; 2 Laboratory of Neurosciences and Mental Health, Petrópolis Medical School, Arthur Sá Earp Neto University, Petrópolis, Brazil

**Keywords:** capitalism, inequality, meritocracy, opinion, social order, sociology

## 1 Introduction

Meritocratic rhetoric is structurally embedded in the political and cultural imaginaries of contemporary liberal democracies, within which the vast majority of modern societies are situated. According to this ideology, through sufficient individual effort, anyone can become a materially and socially “successful” human being. However, this discourse conceals a fundamental flaw: competition can only be considered fair if the initial conditions among all competitors are equivalent, such that no structural inequality interferes with the process (Young, 1958; Sandel, 2020).

Rather than offering a comprehensive or neutral review of meritocracy as a social theory, this article advances a critical and normative position. It argues that meritocracy functions primarily as an ideological mechanism that legitimizes structural inequalities by individualizing social outcomes. The aim is not to refine meritocracy or to assess its partial applicability, but to demonstrate its conceptual and empirical untenability as an explanatory framework for individual success in real social contexts.

This article examines meritocracy in relation to the natural and historical–cultural realities of human beings and, subsequently, to human diversity itself. We analyze six successive levels at which equivalence among individuals would be necessary for meritocracy to be conceptually and empirically admissible. Anticipating the impossibility of equivalence across these six dimensions, we dismantle the ideological foundations of meritocracy and expose its political function within capitalist societies.

## 2 Meritocracy: concept and political function

The concept of meritocracy originated in Young (1958) critical and dystopian essay, whose explicit intention was to denounce the naturalization of inequality under the guise of individual merit. Young's argument centers on the idea that merit may only be meaningful in highly restricted contexts—such as the military—where participants are subjected to standardized training and relatively uniform conditions. Even in these contexts, merit remains contingent upon institutional design rather than natural fairness.

With the rise of neoliberalism in the late 20th century, however, this restricted logic was ideologically expanded to society as a whole and transformed into a prescriptive doctrine for human performance. Meritocracy became a moral framework through which social hierarchies were justified, regardless of historically produced inequalities in wealth, power, education, race, gender, and health (Bowles and Gintis, 2011; Littler, 2017).

Bourdieu's analysis of symbolic violence is particularly relevant in this regard. As Bourdieu (1998) demonstrates, meritocracy operates by presenting outcomes derived from accumulated social advantages as if they were the product of individual talent and effort alone. In doing so, it obscures the structural mechanisms that organize life chances and transforms historically contingent inequalities into morally legitimized hierarchies.

## 3 The structural impossibility of meritocracy: levels of equivalence

To operationalize this critique, we delineate six dimensions of human development and social organization in which equivalence would be required for meritocracy to function as a valid explanatory principle. The absence of equivalence at any one of these levels is sufficient to undermine meritocratic claims; taken together, they render meritocracy structurally impossible.

### 3.1 Economic equivalence

Research on wealth distribution demonstrates that extreme and self-reinforcing inequalities characterize contemporary capitalist societies. Piketty (2014) shows that inherited wealth systematically concentrates resources, time, security, and social networks within privileged families. These resources enable differential investment in children's cognitive, emotional, and social development (Heckman, 2006). Such advantages are neither randomly distributed nor individually earned; they are cumulative and intergenerational.

Under these conditions, the notion of equal economic starting points becomes analytically indefensible. Meritocratic competition presupposes an economic equivalence that does not exist and cannot be produced without dismantling the very structures that sustain capitalist accumulation.

### 3.2 Cultural equivalence

Beyond material resources, cultural capital plays a decisive role in structuring opportunity. As Bourdieu and Passeron (1970) demonstrate, educational institutions privilege specific linguistic, aesthetic, and behavioral codes associated with dominant social groups. These codes are presented as neutral standards of intelligence or competence, while alternative forms of cultural expression are devalued.

Even when economic conditions appear comparable, cultural asymmetries shape individuals' capacities to navigate institutions and to be recognized as “meritorious.” Moreover, structural forms of discrimination—such as racism, misogyny, homophobia, and ableism—operate as culturally reinforced barriers that profoundly distort the conditions of competition (Almeida, 2019; Sue, 2010).

### 3.3 Educational equivalence

Educational systems do not compensate for social inequality; they largely reproduce it. Access to high-quality education remains unevenly distributed even in countries with substantial public investment in schooling (OECD, 2019). In Brazil, educational trajectories are deeply stratified along class and racial lines, reflecting broader patterns of historical exclusion (Saviani, 2008; Heringer, 2018).

Meritocratic discourse treats educational outcomes as evidence of individual effort, while systematically ignoring the institutional and territorial inequalities that structure access to learning environments. Under such conditions, educational equivalence remains a rhetorical fiction rather than a social reality.

### 3.4 Psychological equivalence

Developmental psychology consistently demonstrates that early-life adversity—such as exposure to violence, neglect, chronic stress, or parental psychopathology—has long-lasting effects on cognitive, emotional, and social development (Cicchetti, 2016). These adversities are unevenly distributed across social groups and are closely linked to economic and cultural inequality.

Although psychological suffering is not exclusive to economically disadvantaged families, access to protective environments and mental health resources is profoundly unequal. As a result, individuals enter competitive social arenas with markedly different psychological dispositions and capacities, further undermining any claim of equivalent starting points (Shonkoff and Garner, 2012).

### 3.5 Biological and health development equivalence

Health conditions from gestation through early childhood vary significantly across regions and social groups. In Brazil and other unequal societies, structural deprivation contributes to malnutrition, exposure to environmental toxins, and limited access to prenatal and pediatric healthcare (Victora et al., 2008). These factors directly affect neurodevelopment and later performance.

Biological development is therefore inseparable from social organization. Inequalities in health are not natural deviations from an otherwise level playing field; they are socially produced disparities that shape individuals' capacities long before meritocratic evaluation begins.

### 3.6 Genetic equivalence

It is crucial to clarify that the discussion of genetic variability does not imply biological determinism or a reduction of social outcomes to innate traits. On the contrary, the argument advanced here is sociological. Human genetic diversity becomes socially consequential only within political and economic systems that selectively reward certain forms of functioning while penalizing others.

Even under hypothetically equal social conditions, the inherent variability in human genetic composition— affecting temperament, vulnerability to disease, neurodevelopment, and physical ability—would preclude any notion of full equivalence (Plomin, 2018). Genetic diversity does not explain inequality; rather, it exposes the normative fiction underlying meritocratic evaluation systems, which require the erasure or devaluation of difference to appear fair, in the end.

Taken together, these six dimensions demonstrate that meritocracy cannot be rendered conceptually coherent or empirically applicable in real social contexts.

## 4 Ableism as a mechanism of inequality justification

Ableism constitutes a central mechanism through which meritocratic ideology sustains itself. As Campbell (2009) argues, ableism assigns differential value to individuals based on normative assumptions about bodily and cognitive functioning. Within meritocratic logic, deviation from these norms is framed as a personal deficiency rather than as an expression of human diversity.

Meritocracy thus presupposes an idealized model of the human subject: efficient, productive, autonomous, and endlessly adaptable. This model is neither universal nor attainable, yet it becomes the implicit standard by which all individuals are evaluated (Garland-Thomson, 2011). Ableism enables meritocracy to translate structural exclusion into moral judgment, transforming systemic barriers into individualized failure (Wong, 2020).

## 5 Affirmative action, quotas, and redistributive justice

Given the structural impossibility of meritocracy, redistributive policies emerge not as exceptions to fairness, but as necessary responses to inequality. In Brazil, socioeconomic and racial quotas in public universities—established by Law No. 12.711/2012—have significantly expanded access to higher education without compromising academic performance (Heringer, 2018; Carvalho, 2004).

Critiques of affirmative action often rely on the assumption of pre-existing equality and interpret redistribution as “reverse discrimination” (Sowell, 2014). Such critiques disregard the historical legacies of colonialism, slavery, and institutional racism, as well as ongoing processes of cultural reproduction and ableist exclusion that shape real opportunities.

## 6 Final considerations

Meritocracy is a fragile and internally contradictory concept when examined through even the most basic analytical lenses. While it should not occupy a central place in sociological theory, it remains a dominant ideology among policymakers and public opinion leaders in societies marked by inequality.

In the Global South, where colonial histories, racial hierarchies, and/or extreme wealth concentration persist, meritocracy performs an especially pernicious ideological role. It reframes historically produced deprivation as individual failure and transforms structural violence into a moral deficit.

Theoretically, this analysis challenges approaches that treat meritocracy as a flawed but reformable principle. Instead, meritocracy must be understood as structurally dependent on inequality and incompatible with the irreducible heterogeneity of human life. From a policy perspective, redistributive measures, accessibility policies, and universal social protections should not be justified as temporary corrections to a malfunctioning meritocracy, but as permanent responses to human diversity itself.

Human life unfolds within interdependent networks of history, contingency, and social structure—far beyond what can be captured by individualized notions of merit (Rose, 2007; Butler, 2004).
